# General practitioners’ role in safeguarding patients with dementia in their use of dietary supplements. A qualitative study

**DOI:** 10.1080/02813432.2023.2283182

**Published:** 2024-02-07

**Authors:** Hilde Risvoll, Torsten Risør, Kjell H. Halvorsen, Marit Waaseth, Trine Stub, Trude Giverhaug, Frauke Musial

**Affiliations:** a NAFKAM, Department of Community Medicine, UiT, The Arctic University of Norway, Tromsø, Norway; b NLSH Vesterålen, Department of Neurology, Stokmarknes, Norway; c Valnesfjord Helsesportsenter, Valnesfjord, Norway; d Section for General Practice, Department of Community Medicine, UiT, The Arctic University of Norway, Tromsø, Norway; e Section for General Practice & Research Unit for General Practice, Department of Public Health, University of Copenhagen, København K, Denmark; f IPSUM research group, Department of Pharmacy, UiT, The Arctic University of Norway, Tromsø, Norway; g Center for Profession and Quality, University Hospital of North Norway, Tromsø, Norway

**Keywords:** General practitioners, dementia, dietary supplement, patient safety, qualitative research

## Abstract

**Objective:**

The use of dietary supplements (DS) may cause harm through direct and indirect effects. Patients with dementia may be particularly vulnerable. This study aims to explore general practitioners’ (GPs’) experiences with DS use by these patients, the GPs perceived responsibilities, obstacles in taking on this responsibility, their attitudes toward DS, and suggestions for improvements to safeguard the use of DS in this patient group.

**Design:**

Qualitative individual interview study conducted February - December 2019. Data were analysed using systematic text condensation.

**Setting:**

Primary healthcare clinics in Norway.

**Subjects:**

Fourteen Norwegian GPs.

**Findings:**

None of the informants were dismissive of patients using DS. They were aware of the possible direct risks and had observed them in patients. Most GPs showed little awareness of potential indirect risks to patients with dementia who use DS. They acknowledged the need for caretaking of these patients. Although there were differences in practice styles, most of the GPs wished to help their patients safeguarding DS use but found it difficult due to the lack of quality assurance of product information. Furthermore, there were no effective ways for the GPs to document DS use in the patients’ records. Several suggestions for improvement were given by the GPs, such as increased attention from GPs, inclusion of DS in the prescription software, and stricter regulatory systems for DS from the authorities.

**Conclusion:**

The GPs had initially little awareness of this safety risk, but there were differences in practice style and attitudes towards DS. The GPs did not perceive themselves as main responsible for safe use of DS in patient with dementia. The most important reason to disclaim responsibility was lack of information about the products. One suggestion for improvement was better integration of DS in patients’ medical record.

## KEY FINDINGS

Currently, little is known about general practitioners (GPs) caretaking of patients with dementia who use dietary supplements (DS). Our study showed that:

The GPs in this study showed little awareness of the potential safety risk that DS use may represent for patients with dementia.

Several obstacles in the treatment setting and in the regulation of DS make it difficult for the GPs to assume full responsibility for patients with dementia who use DS.

Lack of evidence about DS safety and effect adds to professional uncertainty and may cause frustration or reluctance to address the issue.

## Introduction

DS are defined by The United States Dietary Supplements Health and Education Act of 1994 as products meant to supplement the diet. Included are vitamins, minerals, herbs, botanical products, amino acids, and dietary substances [1]. Some DS are vitamins with clear recommendations regarding indication, dosage, and monitoring to correct a diagnosed deficiency. Others are composite products containing components from more than one natural source (herbs, herbal extracts, vitamins, fatty acids, and so forth) in combination. The regulation of production, sale, marketing, and use of DS is limited compared to prescription drugs (PD) [2]. DS are often used in an attempt to improve general health [3,4], but also to improve specific conditions such as dementia, even though there is no evidence of documented effect [5–8]. In their national guideline for dementia, the Norwegian Directorate of Health advises against using DS for dementia symptoms [9]. Nevertheless, studies from Norway as well as from Germany and Australia show that up to 57% of patients with dementia use DS [10–12]. The use is often not disclosed to GPs or other healthcare personnel [10,13,14].

The use of DS may compromise health. A direct risk, that is associated with the product itself, is interactions with PD or adverse reactions such as hepatotoxicity, which in the worst case could be lethal [15]. Hypervitaminosis is another risk [16]. Moreover, cases of illegally added PD to DS have been disclosed, also for products marketed as cognitive enhancement supplements [17]. In addition, DS may impose indirect risks, that is risk related to the condition of use, for patients with dementia [11, 18], such as mixing DS up with PD or taking more DS than recommended [11].

In a former study of 151 patients with dementia, of which 70 used DS, we found possible interactions between DS and PD in 11% of the DS users [11]. This led to an increased focus on and concern about these patients’ safety. In Norway, the majority of clinical encounters with doctors take place in primary care, which is why the role of the general practitioner (GP) in managing this issue is obvious [19]. It is estimated that 100,000 Norwegians have dementia [20]. In what concerns these patients, GPs must always consider their progressive decline in cognitive function, potential lack of judgement, and reduced ability to maintain their own interests including the proper use of both PD and DS.

The efficiency and quality of the Nordic primary care models are documented [19]. Even so, GPs may experience and manage uncertainty and ambiguity as an integrated part of daily work, especially when working under conditions of limited time and resources, and they often experience doubts concerning their clinical decisions [21]. This is especially true when it comes to patients with dementia [22].

The responsibility of DS hangs in the balance between the patient’s concern and the doctor’s responsibility. This becomes especially clear in the caretaking of patients with dementia as dementia may pose challenges to capacity and judgement. DS sustain an ambiguous position in medical practice due to a dual role functioning as a diet and a remedy that promotes health, something the patients take at their own discretion but sometimes expect their medical doctors to monitor.

Some studies have investigated how GPs communicate with patients in general about DS [23]. The present study is to our knowledge the first to specifically explore GPs’ professional practice concerning home-dwelling patients with dementia who use DS.

### Aims

This study aims to investigate the encounters of GPs in relation to the use of DS by patients with dementia. Moreover, we sought to comprehend the GPs perceived responsibilities, their challenges in facing these responsibilities, their attitudes towards DS, and their suggestions for enhancing safer use of these supplements among this patient group.

## Material and method

### Study design

The nature of the research questions is intentionally broad and exploratory, and we have chosen qualitative individual interviews as the research approach to allow both descriptive and exploratory work. Qualitative methods may contribute to a better understanding and improved level of knowledge regarding important health and well-being issues. An interview guide (see Supplementary Material 1) was developed by the authors and the patient research partners (see Acknowledgements) based on the aim of the study and previous research [11,18,24,25]. The interview was piloted by asking the questions from the interview guide to one GP not participating in the study. The goal was to explore whether the questions were relevant to general practice, and to evaluate the time-use. No questions were changed after the pilot.

### Study area and setting

The informants were GPs located in North Norway within the Norwegian primary healthcare system. North Norway is an arctic/subarctic geographical area and sparsely populated with a few larger towns. The area is 112,986 km^2^ and in 2022 there were 483,536 inhabitants [26].

The Norwegian healthcare system is funded publicly although private actors do exist [19], it covers all inhabitants in need of healthcare. In the publicly funded healthcare system, all inhabitants are entitled to adequate help after paying a small fee. The service covers among other things visits to GPs, home care service, and most PD for chronic diseases such as antidementia drugs (reimbursable prescriptions); it does not cover DS. The Norwegian primary healthcare system places GPs in a central role [19]. All Norwegian inhabitants (5.5 million) are entitled to a GP regardless of income, age, ethnicity, geographic affiliation, or disease status (the service is based on the principle of equality). GPs have responsibility for patients’ health and safety and organize and coordinate patients’ clinical pathways.

### Recruitment of informants

Based on the public GP index, we invited a purposive, diversified sample of GPs by phone, to cover different groups of gender, age, native/non-native Norwegian according to their names, and a rural/urban workplace in North Norway, see Table 1. We selected only one informant from each GPs office. In smaller municipalities, only one GP was chosen. We initiated the process in a single municipality by randomly selecting a GP. In each subsequent municipality, we intentionally chose a GP of a different gender and age, creating a diverse sample of GPs in terms of age and gender. Furthermore, we ensured a balanced geographical distribution by including a mix of rural and urban municipalities. To promote diversity, we deliberately included a percentage of GPs with non-Norwegian names. It is worth noticing that we excluded GPs whom HR had previously interacted with, except for one case, where the interaction occurred more than 20 years ago. Only one GP refused to participate without further justification. The informants were offered 81 euros, estimated to represent one hour’s working time, for their participation according to the standards of UiT The Arctic University of Norway. Not all informants wanted a compensation.

**Table 1.** Characteristics of the informants (*n* = 14).

Characteristics	Categories	Number^a^ or mean (range)^b^
Gender	Female/male	7/7^a^
Age	40 years	4^a^
	40-55 years	6^a^
	>55 years	4^a^
Birthplace	Norway/abroad	10/4^a^
Medical degree from	Norway/abroad	9/5^a^
Workplace*	Urban/rural	6/6^a^
Work experience as GP	Years, mean (range)	15.5 (1-36)^b^
Practice list size	Number of patients, mean (range)	906 (450-1,500)^b^

* Rural was defined as a municipality of <50,000 inhabitants.

GP: General practitioner.

### Data collection

The interviews with 14 GPs were conducted by HR between February and December 2019. Nine interviews were performed face-to-face, most often in the GP’s office, and five on the telephone. The interviews lasted on average 48 min (range 19-89 min). The interviews were audiotaped and transcribed verbatim. HR, FM and KHH assessed the transcripts consecutively and decided that the study had enough information power after 14 interviews.

### Analyses

The analyses were inspired by the research questions and knowledge derived from former studies [11, 24, 25], including a theoretical model for direct and indirect risk [11]. The data material was analysed using systematic text condensation, a method for thematic qualitative analysis [27]. The analysis followed these steps: (i) reading all the transcripts to obtain an overall impression; (ii) identifying units of meaning and coding for these units; (iii) condensing and summarising the contents of each of the coded groups; and (iv) reconceptualising the data making generalised descriptions and concepts reflecting the GPs management of patients with dementia who use DS. This was done in several rounds for each step by FM, KHH and HR. After step iii, MW and HR read all transcripts to quality check if the findings reflected the opinion of the informants and TG performed a top-down quality control, by reading the preliminary findings before the transcripts. All authors joined the analysis at step iv. The multidisciplinary team behind this study has experience and competence covering a broad range of the healthcare system: general practice, memory clinic, pharmacological, psychological, and caregivers’ expertise, and expertise in complementary and alternative medicine. We have provided information about the authors’ preconceptions in Supplementary Material 2. A bilingual native English speaker assisted in translating quotes into English.

All informants were offered a read-through of their own transcript and were invited to give feedback on the first version of findings. Four informants provided general feedback (e.g. “interesting findings” and “important work”). None suggested any corrections.

### Ethics

All informants gave written informed consent to participate and were entitled to withdraw their consent at any time. All audiotapes were deleted, and the transcripts anonymised at the end of the study. The informants are only referred to by number in the text. Information that could facilitate recognition is left out.

## Findings

The characteristics of the informants are presented in Table 1.

The findings from the interviews were organised into four main themes, see Table 2.

**Table 2.** Main themes and subthemes extracted from the interviews with the GPs.

Main theme	Experience of risks from DS	Self-perception of professional role	External factors challenging the caretaking of patients with dementia who use DS	Suggestions on how to improve safety of patients with dementia who use DS
**Subthemes associated with each main theme**	Direct risk	Attitudes towards DS	Lack of available product information	Specific suggestions for improvements
	Indirect risk related to dementia symptoms	Unawareness of DS use	Lack of time	Ambivalence regarding the automated drug dispensing system
		Unclear lines of responsibility	Lack of sufficient tools	
		Professional role understanding	Insufficient laws and regulations	

DS: dietary supplements, GP: general practitioner.

Several of the informants appreciated the subject of this research project or had more thoughts about it afterwards than before the interview. The main impression was that none of the informants had reflected much, if at all, about the issue of the risk of patients with dementia using DS, and several said they would pursue this issue more closely in the future:

“We probably have a fairly common policy here at the practice, that we haven’t taken it into consideration very much, but now we’ve actually begun to talk about it, we can actually see that DS should be included in a medical overview. So that we do in fact know what the patients are using” (id 10).

Each of the GPs had relatively few patients with dementia (from 3 to 30) on their patient list. The GPs stressed the importance of working with the home care service/memory team and relatives in the follow-up.

## Risks from DS use

### Direct risks

Most of the GPs had observed that use of DS could constitute a direct risk for patients’ health. Examples were elevated liver enzyme tests, increased creatinine or creatine kinase levels and INR (international normalised ratio) tests, changes that normalised when discontinuing the DS. In the same manner, DS had caused dizziness, lethargy, malaise and vomiting in some patients including patients with dementia.

“There was a recent example, last week, when a lady of 88 came and she had begun to use a DS but had a bad physical effect from it (…). She felt weak. She felt a bit out of it, in fact. (…) We looked into it, and she was never to use it again. (id 5)

One GP had reported side effects from DS to RELIS (the Norwegian national network of four regional medicines information and pharmacovigilance centres). Unlawfully added oestrogen, caffeine and narcotics in DS were mentioned (patients with dementia were not mentioned specifically). Several had been contacted by pharmacists and warned of interactions between DS and PD relating to specific patients. Regarding patients with dementia, GPs recalled the use of fat-soluble vitamins over the recommended dose, and symptoms of the serotonin syndrome due to interaction between Escitalopram and St. John’s wort.

### Indirect risks relating to dementia symptoms

GPs were aware that although a regular diet usually provides enough vitamins and minerals, patients with dementia may need supplements due to inadequate food intake.

The use of DS may result in mistakes because of the many tins and boxes at home causing patients with dementia to lose track of what they took and why. The boxes of DS can be mistaken for PD and vice versa. One of the GPs said that it was impossible to judge whether the products were effective or not without discontinuing and evaluating afterwards.

Some informants had patients with dementia who had mixed up PD with DS because the names were similar and who subsequently stopped taking the PD. One patient with vascular dementia preferred to use an oats-based DS, rather than statins. There was also a concern that patients with dementia used more DS than recommended.

“I soon became worried about whether people with dementia might mess with what they take. Are they taking more than they should? “(id 11).

Caretaker/next of kin, home care service and pharmacies could address concerns about the possibility of making mistakes to the GPs.

“I’ve had episodes where the home care service has asked for the DS to be included in the list of medications. This is because they’re worried about there being so many boxes and stuff. They want to get an overview. What the patient **should** have and **shouldn’t** have” (id 7).

Some patients could have difficulty declining telephone sales of DS or cancelling DS subscription and end up buying more DS than intended. Relatives could be concerned about economic exploitation. Several GPs suggested that the DS industry exploited patients’ health anxieties, and that advertising played on this.

GPs’ self-perception of their professional role including attitudes towards DS and knowledge about DS. Taking lots of tablets, especially large ones, affected elderly debilitated patients’ appetites and contributed to malnutrition

### Attitudes towards DS in general (all patients)

None of the informants was dismissive of patients using DS, but several expressed a certain scepticism, as DS may cause harm, are expensive or have limited effect, if any. One GP did not want to deal with DS in the current situation where there is a lack of exact information; at the same time, this GP, and several others, wanted an overview of DS which could have positive effects, in order to advise the patients. One GP had a consistent curiosity and positive attitude to herbs, and at least two of the GPs were positive about recommending herbs which were safe. None of the informants had experienced conflicts with patients or relatives caused by DS. Placebo effect was claimed to be beneficial for patients, especially for disorders with no medical cure. Some of the GPs were also open to the idea that certain DS products in fact, although not documented, could have a therapeutic effect beyond the placebo effect. Respect was voiced for patient choice and self-determination:

“If people believe in it and it actually works, why run it down as long as it’s not dangerous” (ID 8).

### Unawareness of DS use

Patients’ DS use was not a central part of the clinical practice, and the informants did not follow patients with dementia more closely than other patient groups on this point. The GPs had a variable focus on DS, from having “parked it”, or scarcely remembering situations where this came up, to having many thoughts about this. One GP reported systematically questioning patients about DS use. This GP believed that approximately 50% of old patients and patients with dementia used DS. The GPs who did not ask systematically believed that 5-10% of these patients used DS. Certain “red flag situations” led to GPs asking about DS use, e.g. warfarin treatment, elevated liver enzymes and diffuse symptoms in the elderly. Most often a conversation about DS was prompted by an enquiry from the patient, relatives, or home care service. Some GPs did not generally inquire about DS, apart from vitamins. Several GPs thought that they generally did not know which DS their patients used, regardless of these patients having a diagnosis of dementia or not.

“And **then** it can happen that we’re not clear about what they’re actually using because, well, sometimes we’re a bit too indolent to ask” (id 10)

The use of DS might be discovered when a patient with dementia moved out of their home and all the containers were found. One GP thought that many composite products could slip under the radar as “my vitamin pills”.

“It’s also been the case that someone from home care service has got in touch because they can see loads of boxes and different things lying around on the kitchen table and they wonder what they are” (id 4).

Several GPs mentioned that communication was essential to ascertain the use. It was important to have a non-judgmental attitude towards patients who used DS but rather to ask about the patients’ motives, to be “*someone patients wanted to consult with”* (ID 4, 9). Several said they thought that patients might refuse to discuss their use with them because they were afraid of being blamed, ridiculed, not being heard, or that it was not relevant. Some GPs consciously tried to hide their scepticism, to ensure communication, and to show in many ways that they did not solely focus on PD but were open to discuss other methods of improving the patient’s health.

“We need to provide information about this in a sober manner, but it’s also important to guard against being like a watchdog, because then they won’t dare raise it with us. I think that’s part of the reason it doesn’t always get brought up: I think a lot of patients think ‘he’s a doctor, he’ll be sceptical about this’. I try to keep my scepticism to myself, you know? I’d rather hope they’ll open up and tell me about things” (id 7)

### Unclear lines of responsibility

A central question was whether patients’ DS use was part of a GPs job. Unlike PD, which quite clearly is the GP’s responsibility, DS were regarded as the patient’s own responsibility by all the GPs. It was emphasised that patients make their own choices. The only exception was DS specifically initiated by the GP. On further questioning about whether this also applied in dementia cases, all the GPs accepted the need for more responsibility for safety reasons. Some of the GPs also accepted assessments of DS in general (for all patients), but not the responsibility.

“I actually feel I **should** take quite a large responsibility for this because it may have implications for medications I have prescribed, and overall health, but I have to admit I haven’t taken that responsibility” (ID 10).

“For those who have dementia and cannot understand relevant information, then the responsibility is more on us” (ID 2).

Responsibility for the use of DS by patients with dementia did not seem to be an issue any of the GPs had considered before. The GPs did not want the sole responsibility, nor the primary responsibility for safeguarding DS use in these patients, especially if they had not initiated its use. Instead, they felt that the relatives, who often had bought these products, should be more responsible. However, ambiguities relating to this responsibility clearly existed, as GPs perceived that not all patients or relatives understood the potential risk of using the DS. The GPs also saw home care service as responsible.

“I think that investigating DS use would be worth doing to a greater extent than I do at present. But I also think that those with a declared cognitive impairment, they rarely buy things themselves, and they rarely pay their own bills. And so I think the caregivers who buy things for them have a responsibility. Most things are the GP’s responsibility, in a way, but at the same time I think that the GP’s responsibility is so broad that it has to be limited to some extent, so that we don’t have the primary responsibility for this. (id 4)”.

The GPs restricted their responsibility to identifying use and possible risks, and after that to giving advice. Any responsibility beyond that was less clear. The GP who asked patients systematically about their use of DS, also tried to evaluate whether the products were effective or not. Generally, the usage then ended. Several took, or would take when relevant, the initiative to discontinue harmful products with the help of relatives or home care service.

A lack of available, reliable information on effects, safety profile and sometimes also DS contents was the main reason that this responsibility was perceived as problematic.

One GP considered:

“We must deal with the reality that people use a number of things which affect the medicines we prescribe. So, if we refuse to deal with that, then that could even be dangerous for the patients” (ID 9).

### Understanding of the professional role

Two attitudes toward the understanding of the professional role emerged in the interviews, differences in risk assessment and different value balance between evidence-based and experience-based knowledge.

### Uncertainty/risk assessment

Lack of access to valid information about individual DS products caused a feeling of uncertainty. The GPs therefore deemed it difficult to talk to patients about this. They ended up preferring to say, “*I don’t know about this, but it doesn’t seem risky for you*” (ID 2/3/4/6/7/8/10/13/14) “you can use it, *if you can afford it*” (ID 3/7/13). The GPs used the term “advice” rather than “recommendation” regarding DS and several pointed out that “*my saying that you can use this doesn’t mean I recommend it*” (ID 2/3/4/6/7/10/12).

“The whole problem is that I don’t have a proper assessment basis. It’s exceedingly difficult, since I can’t find any quality-based information about whether ginkgo biloba has any interaction with a specific medication. I don’t feel comfortable with that” (ID 12).

Others thought that if they did not identify concrete safety threats relating to the specific product, then patients could try and see how it went.

“I’m not sure I can find secure knowledge about it, but that’s not so important to me, as long as the actual patient experiences a positive effect and there aren’t any noticeable side effects” (ID 11).

Whether this discrimination in risk assessment reflected their medical practice in general did not emerge.

### Evidence-based knowledge versus experienced-based knowledge

Most of the GPs mentioned evidence-based medicine as a gold standard, and that most DS have no evidence-based proof. A few of the GPs mentioned other sources of knowledge as being important when it comes to judging the effect, like historical evidence, personal experience, and the patient’s experience (experience-based knowledge). However, the majority judged anecdotical evidence as insignificant, but believed patients found these types of proof most important.

“*Patients prefer anecdotal evidence, a face, someone they know, to scientific studies*” (id 7).

Comprehensive caretaking, including diet and lifestyle, were an important part of practice. Some of the GPs realised during the interview that this comprehensive caretaking should also include DS to a greater extent than was currently the case.

“I’m really interested in natural functions like sleep and diet, and of course the use of drugs, smoking, and alcohol. So that’s all part of an anamnesis, it’s just that my awareness of dietary supplements **specifically,** has not been quite as great as I now realise it should have been. Particularly regarding those who are cognitively impaired” (id 1).

On the other hand, one GP thought that only medical issues belong in a doctor’s consultation, and not “medicalisation” [28]. One example of medicalisation could be follow-up of DS, “where only quasi-knowledge is available”.

“Medicalisation, in the sense that sectors of society which have nothing to do with health, or with medical terms, are being included under medicine (…) and that we should take it upon ourselves to say something about a specific dietary supplement, of which I have absolutely no knowledge – that’s not my place.” (id 6).

## External factors challenging the caretaking of patients with dementia who use DS

### Lack of available knowledge

Most GPs initially said that they remembered little or no teaching about DS in their medical education or training. What they remembered best was that DS could cause negative interactions with PD. One GP remembered that the doctor’s responsibility to ask routinely about the use of DS was emphasised in training. None of the informants mentioned that DS had been discussed regarding vulnerable patient groups, e.g. patients with dementia.

Several informants stated that relevant national medical journals contain only anecdotical articles on DS. None of the informants had attended continuing education courses where DS was mentioned. The GPs did not generally receive advertising for DS, apart from vitamins and fatty acids. DS were generally not discussed among GPs.

The GPs who provided richest data about DS had obtained extra information about DS either by writing a master’s dissertation on this topic, by being interested in herbs even before their medical training or had learnt about therapeutic DS use during medical training (outside of Norway).

To find reliable producer-independent information was seen as particularly difficult for composite products with herbs, whilst all the GPs felt that purely vitamin-based products were manageable. Information from Internet searches (Google) was considered unreliable. Even identifying the exact contents of the products could be difficult.

“Sales promotions usually come up first, then maybe an explanation that it’s a decoction or extract from a plant, root, or bark, without specifying the active ingredient. Obviously, the decoction of a plant will contain many ingredients” (ID 4).

Most of the GPs reported that they googled the contents and searched in national interaction databases. Some had also contacted RELIS, and the risk in a specific usage situation had been assessed. Since the GPs often did not find valid studies on the effects of DS, their focus was often limited to the documentation of potential risks . The patients’/relatives’ concern was “is it good for my/their health?”.

“I have very little sense of ownership or control engaging with DS when I haven’t learned anything sensible about it, not during my training or in later life, aside from the minerals and vitamins we use” (ID 12).

### Lack of time

Some GPs suggested that capacity, several pressing issues relating to each patient, and fear of falling behind on their time schedule were reasons why DS were not on the agenda.

“.but we are already overworked and … I think that … if this becomes an extra thing in addition to all the other extra things … that becomes … well … the cherry on the top of the cake [ironic]” (id 2).

Most of the GPs did not bring up time specifically, and some felt they had time to ask about DS in the same way as they already did about diet and tobacco. One said that time had to be viewed in the context of lack of knowledge, and that reliable and easily obtainable information about DS-products was needed. One GP considered it unproblematic to assess DS use among patients with dementia. However, if he had to do it for all patients, it would be too time consuming.

### Lack of sufficient tools

According to the GPs, not all DS were integrated in the GPs’ prescription software/prescription mediator set-up, because these were not included or maintained by the FEST (Forskrivnings- og ekspedisjonsstøtte, Norwegian National Formulary). FEST is the Norwegian medicines agency’s database of drugs approved in Europe and Norway. FEST only includes PD and excludes most DS.

You cannot prescribe DS through the prescription software except for some very few products. That in itself is a limitation.” (id 14).

DS is therefore not automatically included in a patient’s referral letter, not in the discharge letter following a hospital stay, and not in the patient’s automated drug dispensing system. Automated drug dispensing system means that patients get their drugs machine dispensed into one unit for each dose occasion and packed in disposable bags [29]. This lack of integration contributed to GPs’ unawareness of what the patients were taking.

### Insufficient rules and regulations

Several GPs were frustrated by the rules and regulations in the field of DS, including no requirement to document claimed effect. Governmental authorities were credited with the responsibility for communicating reliable information on all DS sold in Norway.

The Health Authorities should take responsibility for the public receiving information about the dangers of dietary supplements” (id 4)

## Suggestions for improved safety of patients with dementia who use DS

### Specific suggestions for improvement

Several informants argued that GPs should be more focused, e.g. asking about DS in red flag situations, and a few said that the home care service should contact the GP if they observed DS which were not registered in the prescription software in the homes of patients with dementia.

Although many GPs thought there were too many guidelines already, several envisaged a guideline with an overview of DS which could be safely recommended based on existing documentation of their contents, indication, expected effect, dosage, and potential side effects.

Several thought it would be an advantage if patients’ use of DS could be incorporated into the prescription software. It would provide better overview, but also facilitate digital interaction searches and easy conveyance of information about the patients’ use between different levels of healthcare. An integration could also increase the sense of responsibility.

“If DS became automatically part of the prescription process, it would definitely be labour-saving, because then the interaction search would happen, like, straightaway” (id 10).

The GPs wanted stricter legislation regarding marketing and sales of DS. They also wanted greater control with product quality, including a correct table of contents for active ingredients and quantities.

“It should be against the law to sell DS products in Norway unless the list of ingredients is openly available. And there should be **one** website where you can check everything sold in Norway, where details of the contents are readily available” (id 9).

Randomised control trials on effect were welcomed. One GP recommended that pharmacies should not be allowed to sell DS which lacked documentation of effect or safety.

Some thought information campaigns encouraging DS users/relatives to discuss this with their GP would be useful. Others opposed this due to time pressure or because GPs cannot provide documentation on the effect and safety as this is non-existent for many products. In order to save time, it was suggested that nurses could obtain an overview of wich DS the patients’ currently were using.

### Ambivalence regarding automated drug dispensing system

When asked whether all the DS used regularly by a patient with dementia should be included in the automated drug dispensing system to avoid incorrect dosage and clutter, the GPs were ambivalent. They wanted to avoid patients taking an incorrect dose, but at the same time they had to be able to vouch for what was listed on the prescription card. If no studies of effect/risk were available, this would be difficult. In the case of indication, if the GP had initiated treatment, or if the products were included in the prescription software, the GPs would accept the DS to be included in the automated drug dispensing system. Some said they could include DS if there was no information about negative interactions. There was uncertainty as to whether DS were placed in the automated drug dispensing system at present. One GP mentioned that cost was an issue.

“It’s really a question of costs/usage. And it’s clear that if DS should be in the automated drug dispensing system, then I’d definitely be estimating monthly expenditure and bringing that up with the patient and perhaps the next of kin, if appropriate” (id 13).

## Discussion

### Main findings

The informants’ awareness of the potential of DS-associated harm in patients with dementia was not prominent. They were also in charge of few patients with dementia. The issue of patients with dementia as a vulnerable group had never been brought up as especially relevant related to DS use. However, the informants had observed risks from DS in patients with dementia. After having reflected on the possible safety risk these patients may experience from using DS, most of the GPs in this study accepted a responsibility for this patient group. For competent patients the GPs thought of DS as the patient’s own responsibility.

Generally, DS accounted for a small part of the GPs workday. DS were hardly discussed at all in the relevant fora of medical knowledge development (medical school, medical journals, medical conferences et cetera).

The GPs’ professional practice varied from avoiding discussing DS, to more actively seeking information about patients’ DS use and even adjusting their attitude to be informed about such use, possibly according to how they perceived their professional role in general.

An important reason why GPs had problems keeping track of patients’ DS use, was the lack of appropriate tools in the electronic patient record. The inability to register DS in the medical record was one of the informants’ most important suggestions for improvement of these patients’ safety.

Although the GPs were aware of the potential harm from DS, they experienced a lack of valid information about some of these products which made it difficult for them to give specific advice about effect and safety. This was an important reason why they found it difficult to take responsibility for the safety of patients with dementia who use DS. It seems like the frustration some of the GPs expressed, and for some also a reluctance to address the issue, may be a result of a weakness in the system. The problem involves the definition of DS as merely diet, the lack of authorities’ control and/or regulation and thus a lack of information and documentation on safety and effect.

### Findings in relation to other studies

We have identified some studies involving GPs’ caretaking of DS in patients in general (not only patients with dementia), and some involving other healthcare professionals’ caretaking of persons with dementia who use DS. In two previous studies, both pharmacists and employees in home care service attributed the greatest responsibilityto GPs for the safe use of DS by patients with dementia [24, 25]. The GPs attributed a greater responsibility to home care service and to caregivers than to themselves.

Our results on GPs awareness of risks and benefits related to DS use only partly corroborate a quantitative study investigating 200 UK healthcare professionals’ (GPs, old age psychiatrists, and geriatric nurses) beliefs about the use of vitamin and herbal extracts by persons with dementia [30]. They found that 60% of doctors (GP, old age psychiatrists) agreed or strongly agreed that vitamin and herbal extracts could result in adverse effects and interactions with PD, and 36% believed that vitamin and herbal extracts could have an important role as adjunct therapy in treatment of dementia [31]. Tabet et al. did not disclose their study period, but the paper was published in 2011. It is possible that beliefs about the effects of DS were more optimistic at that time, although later studies have failled to shown positive effects from DS that were initially advertised as promising. The majority of our informants believed DS to have no beneficial effects to counteract dementia and were aware of the potential negative effects. A scientific approach toward DS were most common amongst GPs, but they were open to the placebo effect, and a few were open to additional therapautic effects. For patients, feelings like hope or phenomena like the placebo effect may sometimes be more important than scientific evidence. As an informant said, “If people believe in it and it actually works for them, why run it down as long as it’s not dangerous?”.

All the informants discussed DS with their patients, although several noticed that this happened infrequently, and some were reluctant to have these discussions. Tarn et al. evaluated 1,477 GP consultations in Southern California in 2009–2010 [23]. DS were discussed in a quarter of the consultations. The most common issues (in descending order) were correct administration, potential risks, effectiveness and cost/affordability. Ciba et al. found that a quarter of 515 medical doctors (in specialised healthcare) had received information about adverse reactions of DS from their patients [4]. This may imply that use of DS and potential adverse reactions may be more common than indicated by some of our informants.

Djuv et al. found that only a quarter of Norwegian patients recruited from a GP’s office disclosed their use of herbs to their GP [13]. Several studies have shown that the most common reason for non-disclosure of DS use is that healthcare personnel do not ask [4,14]. GPs’ practice style affects patients [31] and clinical decision making varies between medical doctors even in comparable situations [21,32]. This includes medical tasks that are not done, such as not obtaining sufficient anamnestic information and sufficient medical examinations as shown in another study [33].

The informants had varying views on their own professional role as a GP. Some were active and asked about patients’ DS use and initiated systematic follow-up of these patients. Others were more passive and viewed themselves more as a consultant, limited to answering questions regarding DS use when the patient specifically asked about it. The GPs who appeared to ask more frequently about DS, also mentioned making an effort to be informed by holding back scepticism and showing interest. The GP who asked systematically about DS, found use in half the patients with dementia. GPs who did not ask about this believed that only a few percent used DS. One GP did not want to answer questions about DS since scientific knowledge about effects and risks often does not exist. The most important reason for non-disclosure of DS is not being asked. This fact makes the passive practice style (not asking) less safe regarding adverse effects and interactions with PD

The informants underlined the need for an evidence base for DS. The lack of available information on effect and safety, in some cases also uncertainty about the contents of the DS, was the major reason they felt uncomfortable discussing DS with their patients. In a recent review study from New Zealand perceived lack of evidence, lack of regulation, potential side effects, interactions with PD, and cost were the GPs (*n* = 884) most important concern against complementary and alternative medicine in general [34] indicating that this might be relevant in a global perspective.

The main reason why the GPs found it difficult to deal with DS professionally, was lack of available valid information about the products, which on a deeper level represents uncertainty. Uncertainty is a subjective, cognitive experience of people. The defining feature of this state of mind appears to be the lack of knowledge about some aspects of reality. Especially when the likelihood of risk is unknown, lack of knowledge promotes pessimistic appraisals of risk as well as avoidance of decision-making [35,36]. Uncertainty is known to affect medical practice [21]. One problem in dealing with this uncertainty, may be the lack of communication between GPs regarding DS. The informants said that the topic never came up. One way of dealing with professional uncertainty is discussing difficult topics with peers and adjusting the practice accordingly, as a common standard is set.

Another explanation for the different professional approaches towards DS may be variation in the value balance between evidence-based medicine and patients’ experiences. In this perspective, the use of DS is self-management and patient empowerment [37]. How these values are balanced can differ among healthcare professionals, although the medical training generally values evidence-based medicine more highly [38]. The overall impression of the GPs was that they all expressed the importance of following evidence-based medicine. However, some of the GPs emphasised patient experience and tradition. The GP who followed-up DS use most thoroughly mentioned several limitations to evidence-based medicine, the most relevant in this context is that persons with dementia are generally excluded from clinical trials. This GP also mentioned that evidence-based recommendations based on clinical trials sponsored by pharmaceutical companies can be less trustworthy, because economic interest can influence results. Some authors have supported this view [39,40].

With some exceptions (e.g. vitamin supplements for deficiencies), DS generally falls outside evidence-based medicine. In a German qualitative study, GPs (*n* = 20) who practiced CAM (complementary and alternative medicine) therapy expressed a strong focus on helping the individual patient, a strong belief in one’s own clinical experience; and appreciation for the placebo effect [41]. Our informants also supported the placebo effect of DS, and some were open to the idea that some DS may have genuine positive effects even though there is currently no scientific proof of that.

### Strengths and limitations

We believe the multidisciplinary background of the research team has increased the quality and relevance of the interview questions and the interpretation of the findings. To further enhance credibility, we applied investigator triangulation, method triangulation and member check.

The effort made to select a purposive sample was successful and ensured transferability. Our informants covered a broad range of GPs, including geographical diversity. Only one of the GPs was vaguely familiar to the first author before the interview. The participants were recruited by phone by the first author who is also a medical doctor, however not a GP. This might have caused a higher response rate than if contacted by a person with another profession.

We obtained rich data in both face-to-face and telephone interviews; however, interviewing by telephone may have caused loss of some non-verbal information. Neither information about contextual data and facial expressions, nor body language was included in the analysis.

Our study represents exploratory research conducted to help understand a particular topic. The findings cannot be generalized because of the exploratory nature of the study. This qualitative study explore these North Norwegian GPs’ views and practices regarding DS use by patients with dementia. Practices and attitudes are not necessarily transferable between different cultures. The safety question that this study raises is nevertheless universal.

The GPs were offered economical compensation according to the University’s standards to partly compensate for the time they spent being interviewed. A potential influence on the informants’ answers cannot be ruled out but is considered unlikely. Some informants even declined the offered compensation.

### Significance of the study

GPs can uncover DS use by patients with dementia as part of their job. Although all the informants wanted to help their patients, this study revealed a lack of attention which may represent a general attitude among medical doctors towards the safety aspect of DS. According to the informants, the topic almost never comes up in fora where GPs gain medical knowledge, except during medical studies in lectures covering DS-PD interactions. The topic of patient safety related to DS use needs to be addressed, and the medical education should highlight both the responsibility to uncover such use, and the need to secure vulnerable patient groups, such as patients with dementia. Since patients do not always disclose DS use, the GPs need to ask about such use to be informed. According to the GPs, they collaborate with home care service and sometimes with pharmacy employees about DS in a non-systematic fashion. A systematic collaboration to secure the DS use by this patient group would be a huge advantage, but a clarification of each profession’s role and responsibilities seems necessary. GPs, employees in pharmacies and home care service are all healthcare professionals who, as part of their job, can discover use of DS by patients with dementia [24, 25]. Caretaker/next of kin can in many cases contribute to safer use by providing information about use and/or helping to terminate use of unsafe DS products. If the DS use is continued, how should safe administration be ensured? The GPs in this study had no suggestion for safe methods to ensure proper administration of DS to patients with dementia, and they did not want DS without valid information about safety, efficacy, and content to be delivered by the automated drug dispensing system. This is in line with pharmacy employees [24] and some of the employees in home care service [25] who were asked similar questions. This is therefore a topic that needs to be addressed. If none of the central healthcare professions see caretaking of patients with dementia who use DS as their responsibility, this responsibility is in practice left to these vulnerable patients themselves, unless the patients have a next of kin to help them.

According to the informants, there are several hindrances for the GPs to take on this responsibility, for instance lack of awareness of the topic, inadequate tools in the electronic patient records, and especially the lack of valid information about content, safety, and effect of many DS. The GPs suggested that integrated information about DS in the patient’s medical record/prescription software could provide the opportunity for automatic data analyses of potential interactions between DS and PD. Moreover, they suggested that an integration would prompt the GPs to inquire about patients’ DS use and thus increase the feeling of responsibility. Several of the informants had experienced that specific DS caused harm to their patients, so monitoring DS use is an imperative start in securing patient safety. It is also important that information is passed on between the different levels of healthcare service (information transfer at hospital admission), and having the relevant DS registered in the patient’s medical record, will facilitate this.

The availability of valid information about every marketed DS is a precondition for safe use. Only the health authorities can demand documentation of safety, effect, and correct content for all marketed DS. This would require regulatory amendments. Attention must be drawn towards the complex organisational- and system-level mechanisms responsible for creating and maintaining a situation where DS remain in the grey area between food and medicine.

The Norwegian legal regulations on DS are under revision. We recommend regulations that enforce stricter control and that take into account the known or unknown risk/benefit profile of DS. In addition, we see a need to clarify the different healthcare personnel’s responsibilities regarding DS consumption, considering vulnerable patients such as patients with dementia.

## Conclusion

The safety risks from DS in patients with dementia were not issues the informants had considered previously. The interviewed GPs had, however, observed safety problems regarding DS use, including indirect risks in patients with dementia. Although they expressed some scepticism, the GPs were not dismissive of their patients’ DS use. The assessment of this use posed a challenge for the GPs, and they did not consider it their primary responsibility. They generally acknowledged the problematic situation and expressed their wish for available appropriate tools to support their caretaking of these patients. This includes quality assurance information about all DS sold in Norway and prescription software capable of integrating DS into patients’ medical record. The study indicates that increased awareness of this issue among GPs could contribute to improved safety of DS use for this vulnerable patient group in the future.

## Ethics

The Regional Committee for Medical and Health Research Ethics had no objections to the study design (2016/1775). As no patients were included, the project was defined as “quality assurance”. The study was approved by Norwegian Centre for Research Data (2019/357669).

## Supplementary Material

Supplemental Material

Supplemental Material

## Abbreviations

GP: general practitioner
DS: dietary supplements
PD: prescription drugs
