# Supplementary material for: General practitioners’ role in safeguarding patients with dementia in their use of dietary supplements. A qualitative study
Source: Scand J Prim Health Care. 2024 Feb 7;42(1):16–28. doi: 10.1080/02813432.2023.2283182 (PMC10851825; doi:10.1080/02813432.2023.2283182)
Supplement: Supplemental Material [file IPRI_A_2283182_SM1371.docx]

# Semi structured interview guide general practitioners (GPs) (translated and modified)

## Opening questions:

How many of your patients have dementia?

Can you describe how you assess your patients with dementia?

Possible follow up questions:

Do you involve caregiver/home care service in the assessment? *

Is the assessment systematic? *

Do you use guidelines?

What is your opinion on guidelines in general?

Can use of guidelines interfere with your professional autonomy?

Can use of guidelines make it more difficult to treat each patient as an individual?

## Main questions

How do you understand the term dietary supplements (DS)? ( for clarification of the term)

How often do you ask your patients with dementia about their use of DS in order to secure this use?

How do you assess use of DS among your patients?

Possible follow up questions:

What do you do to improve the safety of patients with dementia who use DS?

Does your assessment of patients with dementia who use DS differ from the assessments of cognitive healthy patients? Please explain how.

Do you conduct home visits to these patients?

How often do patients ask you about DS? Which issues are addressed? *

Do you address DS use when you talk about prescribed medication or when you discuss their lifestyle? *

Can you give an example of adverse events or interactions that have happened because a person with dementia used DS? If not, can you give examples from other patients?

How do you handle, or how would you have handled, adverse events or interactions in patients who used DS?*

Can you provide examples of how you, caregivers, or patients have raised the issue of DS use by these patients? *

Have you had positive or negative experiences with DS in your professional career? Please explain. *

How do you secure that your patients with dementia administer their dietary supplement correctly (use of the automated drug dispensing system)?

If you do not ask your patients about DS as part of your routine assessment, what is the reason for this?

Can you give examples of ethical dilemmas you have found yourself in that are related to patients with dementia who use DS?

Have patients’ use of DS led to any conflicts? *

How can one improve the assessment of patients with dementia who use DS?

Would you like to use a guideline for assessment of patients with dementia who use DS? Why? Why not?

Should DS be included in a central database known as the prescription module/prescription mediator set-up? And in the patients’ medical journal? *

Are DS an issue when you perform medication reconciliation? (or possibly fee-for-service reimbursement)? *

Poster with information for patients hanging in the GPs’ office? *

Measures from health authorities? *

Collaboration between different involved parties? (Home care service, next of kin, nurses, et cetera)? *

What is your understanding/interpretation of the term responsibility? (as in GPs’ responsibility)?

How far does the GPs responsibility reach for patients’ use of DS?

Possible follow up questions:

Do you make other considerations about your responsibility towards persons with dementia than for patients without cognitive impairment? If so, why?

Do you accept the responsibility for the safety of patients’ use of DS? Why/why not?

Regarding patients’ use of DS, which part of this use does not fall under the responsibility of a GP? Please give examples.

Who has the main responsibility for the safety of persons with dementia who use DS? *

Which are the main hindrances for taking the responsibility for the safety of patients with dementia who use DS?

Possible follow up questions:

Can you think of a legal matter that can be a hindrance?

Can you think of an ethical matter that can be a hindrance?

Can you think of a practical matter that can be a hindrance?

In your opinion, where can one best find reliable, scientific information about DS?

Possible follow up questions:

Where do you find information about DS? *

How was your medical education when it comes to DS?

Were you trained in the assessment of DS use during your medical education? *

As a professional, have you received advertising material for DS? *

Have you read articles about DS in Journal of the Norwegian Medical Association /non-medical literature? *

Do you get information about DS on web pages? (Relis/NAFKAM)?*

Have DS been a subject in continuing education or in medical conferences? *

Do you discuss DS with your colleagues? *

How do most GPs feel about use of DS among their patients? Is your attitude different from the majority’s’ in any way? How?

## End Question

Do you want to share something that is important about GPs’ assessment of this patient group; something you have experienced, reflections, or something else?

DS; dietary supplements

* New question developed in the process
