# Supplementary material for: General practitioners’ role in safeguarding patients with dementia in their use of dietary supplements. A qualitative study
Source: Scand J Prim Health Care. 2024 Feb 7;42(1):16–28. doi: 10.1080/02813432.2023.2283182 (PMC10851825; doi:10.1080/02813432.2023.2283182)
Supplement: Supplemental Material [file IPRI_A_2283182_SM1336.docx]

# Supplementary material 2: The authors preconception

TG has worked as a teacher in pharmacy and as a consultant at a drug information center serving health personnel. In both roles searching information and giving advice about the use of dietary supplements was a part of her practice.

KHH is a pharmacist who, for some years, has studied the use of medication and dietary supplement in older adults with special attention towards the quality of such use. In relation to the use of dietary supplements, the regulations are unclear about responsibilities for involved health care personnel. In addition, several supplements inform about possible effects that are highly disputable - from an evidence point of view. With the increasing number of older adults with dementia and cognitive decline, it is necessary to investigate several aspects related to health, nutrition and wellness. I believe that this can be solved interdisciplinary, where the views of different health care professions, alongside with opinions from next of kin and the patients themselves are taken into considerations. The importance of understanding why people use dietary supplements and perspectives about attributed responsibilities by health care personnel is of especial value to ensure the safe use of these products in older adults with reduced abilities to safeguard themselves. Also, with the increasing use of different supplements, there is a necessity to develop systems and regulations that monitor different aspects of such use. To succeed, different health care personnel will need to contribute.

FM are a psychologist with a research background in neuroscience, biological psychology, clinical psychology, clinical trials, research methodology, and alternative treatment / complementary medicine (CAM) and risk related to these interventions. Her first contact with alternative treatment / CAM was when she became head of research at the Department for Complementary and Integrative Medicine at the University Duisburg-Essen, Germany in 2006. From that time on she has worked scientifically within the field of CAM. Her major area of expertise in CAM is the conductance of clinical studies on pain in the non-pharmacological arena as well as the integration of biomarkers into these trials, with the aim to explore the potential biological mechanisms of effect. In 2015 she was appointed the first Norwegian professorship for “Healthcare Research – Alternative Treatment” at NAFKAM, Department of community Medicine, The Arctic University of Norway, UiT.

As head of research at Department for Complementary and Integrative Medicine at the University Duisburg-Essen, Germany, she has conducted or been involved in more than 20 studies involving non-pharmacological interventions, such as acupuncture, cupping, Alexander technique, Yoga, medical leeches etc. Since she was quite unfamiliar with these techniques when she started in the field, she felt that she needed to try at least those techniques that we investigated in clinical trials on herself, before exposing study participants to it. Thus, she has experienced acupuncture, dry and wet cupping, GuaSha massage, massage, osteopathy, and healing as part of her profession as a CAM researcher.

In opposite to that, her research and personal experience with herbs and dietary supplements is limited. Having multiple sclerosis, she does take Møller Tran, vitamin B, and another supplement containing short chain fatty acids. There is scientific evidence for these supplements, which is the basis for her decision to take them.

Her principal approach to research in CAM is that there is no difference to clinical research in the conventional arena. She is a quantitative researcher, and, in her opinion, research methodology must be as sound, and evidence based as it should be the case in any type of clinical research. Possibly even more so, because CAM interventions are usually not performed within a conventional healthcare setting, which may increase direct or indirect risk to patient safety.

Reading through the interviews, she realized, that her focus of attention was influenced by her background as a psychologist. She noticed that she was interested in aspects, possibly relating more to a meta-level beyond the immediate question of risk, although related to it. Such aspects are the “perception and concept of men/patient “, “the doctor-patient dyad/relationship”, the understanding of the “doctors’ responsibilities” and “accept” of the patients’ wishes and attitudes. She believes that she focused on these aspects, because assume that they are related to potential indirect risk for the patients.

HR has worked as a clinician in the field of neurology/dementia/rehabilitation for 25 years. As a clinician in a memory clinic, she often received questions from patients or caregivers about dietary supplements. The questions were about safety and effects and difficult to answer as the information on specific DS were sparse. This led to a contact with TG at the pharmacovigilance center, and the idea for this study as a need to focus more on this safety aspect was discovered. HR has otherwise little experience with DS or CAM.

TR believe that many patients use various forms of dietary supplements, herbal medicines, vitamins etc. This is not something he know a great deal about as family physician, and he hope patients do some research if they take it. When it comes to patients with dementia or others where the judgment and/or memory is impaired, this is something he feel even more uncertain about. He rarely has all the information about what medications the patient actually uses, and it is somewhat unclear where the responsibility to ensure alignment with prescription drugs lie. Maybe with him? He knows that there are inappropriate combinations, but he mostly focuses on the information he has in the medical record.

TS is trained as an acupuncturist and homeopath. She holds a PhD in medicine. Phytotherapy was a substantial part of her training as homeopath, and as a former health care provider working outside the official health care system, she prescribed dietary supplements and herbs to patients daily. However not so often to patients with dementia. Her PhD and post-doc focused on risk and patient safety in the complementary and alternative (CAM) field.

MW is a pharmacist with a PhD in epidemiology and a background from community pharmacy practice. Her interest in determinants of dietary supplements (DS) use started at the pharmacy and continued through her academic training and into research on general population survey data. She has been teaching pharmacy students about DS, particularly herbal supplements, for fifteen years, with a special focus on safety. MW’s preconception of general practitioners’ views on DS was that, as a group, they do not concern themselves with patients’ use of DS and do not consider this a problem that they need to handle.
